# Influence of Electrostatic Field on the Quality Attributes and Volatile Flavor Compounds of Dry-Cured Beef during Chill Storage

**DOI:** 10.3390/foods9040478

**Published:** 2020-04-10

**Authors:** Chen-Chen Xu, Hui Yu, Peng Xie, Bao-Zhong Sun, Xiang-Yuan Wang, Song-Shan Zhang

**Affiliations:** 1 Institute of Animal Sciences, Chinese Academy of Agricultural Sciences, Beijing 100093, China; chenxu30510@cau.edu.cn (C.-C.X.); seulbird@163.com (P.X.); baozhongsun@163.com (B.-Z.S.); 2 Shandong Agriculture and Engineering College, Jinan 250100, China; yuhuijn@163.com (H.Y.); xiangyuanlc@163.com (X.-Y.W.)

**Keywords:** high-voltage electrostatic field, dry-cured beef, color, volatile compounds

## Abstract

The purpose was to investigate the quality characteristics of dry-cured beef with different storage times under a high-voltage electrostatic field (HVEF) condition. The pH, moisture content, meat color, and volatile compounds of dry-cured beef samples treated with HVEF (3 kV) were compared with those of a common refrigerator (CON) at days 0, 3, 7, 10, and 14. The results showed that, compared with CON group, the decline rates of the pH and moisture content of beef and ∆*E* values were lower under HVEF storage condition. From the fingerprints, the 42 volatile compounds identified were mainly aldehydes, alcohols, ketones, and esters. The benzaldehyde, trimethyl pyrazine, and maltol contents in the HVEF group exhibited a dramatic increase after 10 days of storage. Principal component analysis revealed clustering of compound classes, distributed in a separate time. Based on the above findings, we concluded that HVEF treatment could promote color stability and enhance characteristic flavor during the storage of dry-cured beef. These results suggested that HVEF might be applicable for dry-cured meat storage techniques.

## 1. Introduction

Meat products are highly susceptible to oxidative deterioration, which can reduce their nutritional value, shorten their shelf life, and be detrimental to consumer health [1]. Salt is one of the most widely used food additives in the food industry, because it is involved in the development of flavoring and food preservation [2]. Dry curing is an ancient technique that stabilizes fresh meat during the salting process due to the combined effect of salt gain and water loss [3]. In different parts of the world, the most popular dried cured meat products are usually made from beef, such as Spanish cecina [4], Italian bresaola [5], Turkish pastirma [6], and Brazilian Charqui [7]. Because of the lack of a steady heat source, the traditional (in sun and shade) dry-cured process of meat is time-consuming and difficult to use to control moisture content, which may cause undesirable changes in color and flavor and shorten the shelf life of meat products [8]. Chaijan [9] showed that the dry salting method reduced the metmyoglobin and redness index of tilapia meat. Therefore, it is important to find a way to avoid abnormal changes and maintain good quality during the storage of dry cured beef.

The high-voltage electric field (HVEF), as a nonthermal food processing technique, has recently received considerable attention; it is often used in the thawing of frozen meat [10,11,12]. Previous studies have reported the effects of HVEF in reducing losses during thawing and cooking pork tenderloin [13]; preserving food freshness [14,15]; and speeding up the thawing rate of pork tenderloin [13], tuna [16], and rabbit meat [17]. Ko et al. [18] reported that HVEF could inhibit protein denaturation and microbial growth of fish meats. Hsieh et al. [19] found that the shelf life quality of tilapia meat could be extended under HVEF condition. Additionally, the application of HVEF technology may keep meat products from being negatively affected due to the use of lower chemical preservatives [20]. However, according to what we know, few studies have focused on the effects of HVEF under the quality traits of dry-cured beef during different storage time.

In the current study, the main aim was to explore the influence of HVEF treatment and storage time (0, 3, 7, 10, and 14 days) on the physicochemical characteristics (pH, moisture content, color parameters) and volatile compounds of dry-cured *semitendinosus* muscles.

## 2. Materials and Methods

### 2.1. Materials

Ten *semitendinosus* beef muscles (about 24 months of age and 72 h post-mortem) were purchased from a local supermarket. After removing the visible connective tissues and external fat, each muscle sample (0.5 ± 0.05 kg) was stored at −18 °C for 7 days until use. Prior to further normal and HVEF storage, the frozen samples were thawed at 4 °C for 24 h.

### 2.2. Experimental Design and Sampling

There were three steps for salting, the first step was conducted by manually rubbing the meat samples (N = 10) with a salting mixture (*w*/*w*) containing NaCl (3%), sucrose (0.15%), sodium nitrite (0.02%), black pepper (1%), and chili powder (0.3%), followed by a 10 d chilling in a cold chamber (BCD-528WDPF, Haier Group Co., Ltd., China) at 4 °C (relative humidity: 70–80%). Then, the samples were washed with running water, and hung for up to 7 days (relative humidity: 60–65%) at 25 °C. In the final step, the meat samples were then hung in a well ventilated room at 15 °C (relative humidity: 70–80%) to dry for 10 days. After drying, each salted sample was chopped into small pieces (10 cm × 6 cm × 4 cm, mean weight: 100 ± 1.55 g), with 15 slices per treatment. A total of 30 pieces were used for five sampling time (three replications for each treatment and sampling time). Afterwards, the samples were vacuum-packed individually in polyethylene bags (20 × 10 cm^2^) using a DZ-280/2SD device (99% vacuum level).

### 2.3. HVEF Treatment

The experimental setup and temperature monitoring system were the same as that described by Jia et al. [21]. The HVEF was composed of the DC and AC voltage generator, a treatment chamber, a miniature refrigerator, and a real-time monitoring system including temperature and humidity. The voltage was set 3 kV. All the samples were stored at 4 ± 1 °C, except that half of the samples were subjected to the HVEF treatment and the other half samples were retained in a common refrigerator as the control group (CON). The experiment lasted for 14 days and various measurements (pH, moisture content, color, and volatile compounds) were performed on 0, 3, 7, 10, and 14 days of storage time. 

### 2.4. Methods

#### 2.4.1. pH

The pH of each sample was measured in triplicate by inserting a pH meter (Model TESTO 205, TESTO Industry Corp., Lenzkirch, Germany) directly into the meat.

#### 2.4.2. Moisture Content

The moisture analysis was done according to the Association of Official Analytical Chemists (AOAC) method 934.01 (AOAC, 1990).

#### 2.4.3. Meat Color 

The color of dry-cured samples was determined using a portable CR-400 Colorimeter (Minolta Co. Ltd, Osaka, Japan) with geometry of diffuse/8° (sphere 8 mm view) and an illuminant of D65/10°. The L^*^ (lightness), a^*^ (redness), and b^*^ (yellowness) were measured by the instrument. Measurements were taken on three different parts of the meat surface, and their average was calculated. Chroma (C^*^) was evaluated C^*^ = (a^*2^ + b^*2^)^1/2^. The total color difference (∆*E*) was evaluated by using Eq.

∆*E* = [(L^*^ − L^*^D0)^2^ + (a^*^ − a^*^D0)^2^ + (b^*^ − b^*^D0)^2^]^0.5^ (1)

where L^*^, a^*,^ and b^*^ values at day 0 were chosen as reference color values (L^*^D0, a^*^D0, and b^*^D0). 

#### 2.4.4. Volatile Compounds Analysis

Dry-cured beef samples (*n* = 18) for volatile compounds analysis were collected in duplicate at 0, 3, 7, 10, and 14 days. Volatile compounds of dry-cured samples were performed using the Gas Chromatograph - Ion Mobility Spectrometer (GC-IMS) equipment (FlavourSpec®, Dortmund, Germany) in accordance with the method of Yang et al. [22] with some modifications. We weighed 2.0 g of each sample, and each sample was transferred to a 20 mL headspace vial and then incubated at 60 °C for 15 min. Thereafter, 500 L of headspace was automatically injected into the injector with a syringe in splitless mode at 85 °C. Volatiles were separated using a hromatographic column (15 m × 0.53 mm × 1 μm, Restek Co., Bellefonte, PA, USA) working at constant temperature of 60 °C. After injection, the nitrogen gas used as carrier gas. The program was set as follows: 2 mL/min for 2 min, 15 mL/min for 8 min, 80 mL/min for 10 min, and 150 mL/min for 25 min. The analysts were separated at 60 °C in the column and then ionized in the IMS ionization chamber of 45 °C. Nitrogen was also used as drift gas at a constant flow of 150 mL/min. The mass spectra of the selected compounds were compared with the GC-IMS library (Gesellschaft für Analytische Sensorsysteme mbH, Dortmund, Germany).

### 2.5. Statistical Analysis

Statistical analyses were tested using the PROC MIXED procedure of SAS (SAS Inst. Inc., Cary, NC, USA) with fixed effects of treatment, storage days, and their interaction. The meat pieces were selected as random effects. Figures were exported from Origin 9.0 (MicroCal Software Inc., Northampton, MA, USA). Differences between means were considered statistically significant at *p* < 0.05. 

## 3. Results and Discussions

### 3.1. pH 

**Figure 1:**
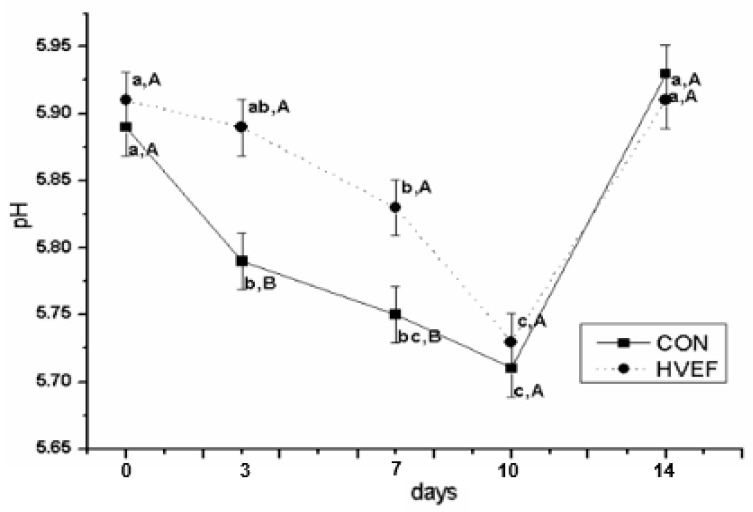
Influence of high-voltage electrostatic field (HVEF) on pH value of dry-cured beef during storage. Different capital letters indicate significant differences (*p* < 0.05) between treatments in the same time and small letters indicate significant differences (*p* < 0.05) between times in the same treatment. Error bars reflect standard errors.

The pH values of dry-cured beef are shown in Figure 1. The pH decreased (*p* < 0.05) slowly with storage time from 0 to 10 days and showed the lowest values on day 10 in both groups. Similar results have been obtained by Guo et al. [23], who reported that as sodium chloride transferring to meat, the increased ionic strength induced changes in the conformation of the protein, which then covered up the basic group in protein, resulting in a decrease in pH. The pH values in HVEF group were significantly higher (*p* < 0.05) than that of the CON group at 3 and 7 days of storage. In this study, the results showed that HVEF had the ability to delay the decrease in pH value and this was in agreement with Ko et al. [20]. This indicated that the integrity of the protein structure of the dry-cured beef stored in HVEF was not disrupted. Although there was no significant difference (*p* > 0.05) in pH value between CON and HVEF groups at day 14, the rate of increase in pH was delayed in the HVEF group. This finding is consistent with previous work demonstrating that an increased pH value might be attributed to the production of basic compounds [24].

### 3.2. Moisture Content 

**Figure 2:**
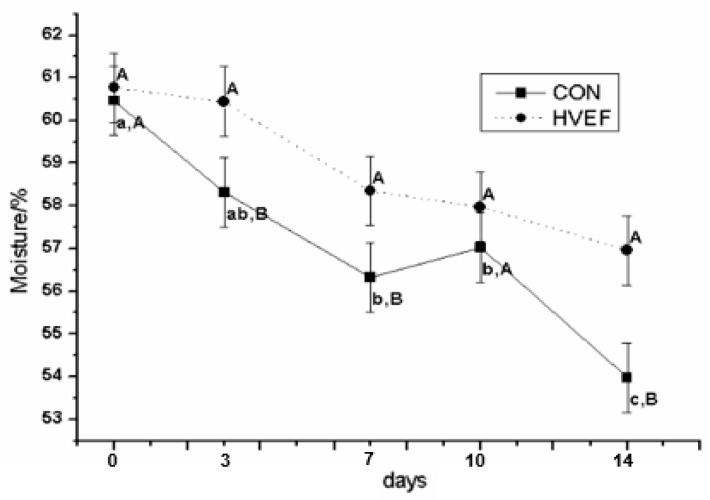
Influence of HVEF on moisture content of dry-cured beef during storage. Different capital letters indicate significant differences (*p* < 0.05) between treatments in the same time and small letters indicate significant differences (*p* < 0.05) between times in the same treatment. Error bars reflect standard errors.

The results of the moisture content of dry-cured beef during storage are presented in Figure 2. As expected, moisture content decreased with storage time, which resulted in the loss of other nutrients. The moisture content in CON group decreased substantially during storage, with the most rapid and significant (*p* < 0.05) decrease occurring from 10 to 14 days. The moisture content of the HVEF group was higher on 3, 7, and 14 days compared with the CON group. Due to heavy absorption of salt, the losses of water in salted meat products were primarily due to immobilized water [23,25]. In addition, it was previously reported that the binding capacity between protein and water was effectively retained under low voltage electrostatic field (2.5 kv) [26]. In the current study, the results showed that enhanced water release during storage was supposed to be the result of protein denaturation, which causes meat products to lose their ability to retain water [27], especially when stored for longer periods.

### 3.3. Meat Color 

The changes in L^*^, a^*^, b^*^, C^*,^ and ∆*E* values of dry-cured beef meat in relation to treatment (CON vs HVEF), storage days (0, 3, 7, 10, and 14 d), and their interactions are shown in Table 1. The stability of the color of dry-cured meat products is a critical quality attribute, as methemoglobin formed by oxidation may cause an undesirable greyish hue [28]. The interaction between treatment and days for color parameters did not show a significant difference (*p* > 0.05). There was not a significant difference (*p* > 0.05) in color values (L^*^, a^*^, b^*,^ and C^*^) between the two treatment during storage. The values of L^*^ and b^*^ tended to rise first and then decrease (*p* < 0.01). The changes in L^*^ value could be attributed to the dark color associated with browning reaction over time in dry-cured meat [29]. While, in the later period, the b^*^ values decreased due to brown melanoids, the product of browning reactions [30]. In this study, the highest a^*^ parameter was observed for the CON and HVEF samples on day 0 (18.45 and 17.17, respectively), while a^*^ and C^*^ values decreased steadily over time caused by myoglobin oxidation, a product of oxidation during storage [24,31]. In addition, the decrease in a^*^ value was likely due to the decrease of nitrosylhemochrome pigment or fading of cured color throughout the storage [32,33]. The value of Δ*E* was significantly (*p* < 0.001) affected by the HVEF treatment and storage time. The determination of Δ*E* showed that the increase in storage time was accompanied by an increase in Δ*E*. This research also observed that the color of CON meat samples was less stable than the HVEF sample (higher Δ*E* value), especially on day 7. 

The results of the current study showed that HVEF treatment could greatly retain the color stability of dry-cured meat without adversely affecting redness, indicating a potential role of high-voltage electrostatic field in maintaining meat-color stability.

### 3.4. Volatile Compounds 

**Figure 3:**
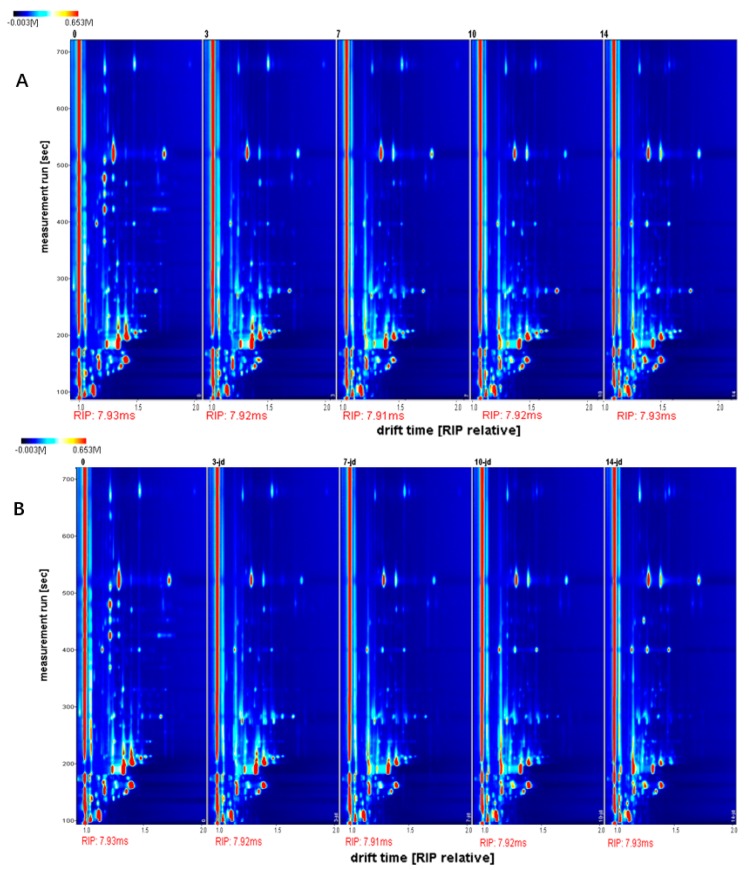
Two-dimensiona topographic plots of dry-cured beef at different times. Storage of dry-cured beef in a common refrigerator is shown in (**A**), 0, 1, 3, 7, 10, and 14 represent refrigeration for 0, 1, 3, 7, 10, and 14 days, respectively. Storage of dry-cured beef in a high-voltage electrostatic field is shown in (**B**), 0, 3-jd, 7-jd, 10-jd, and 14-jd represent refrigeration for 0, 3, 7, 10, and 14 days, respectively.

Figure 3 shows a two-dimensional topographic map of volatile organic components (VOCs) in dry-cured beef during storage. The red vertical line at horizontal coordinate 1.0 is the reactive ion peak (RIP, normalized drift time of 7.92). The spot intensities of VOCs were compared between the two treatments, and we determined which compounds increased, decreased, disappeared, or fluctuated during storage (0, 3, 7, 10, and 14 d).

**Figure 4:**
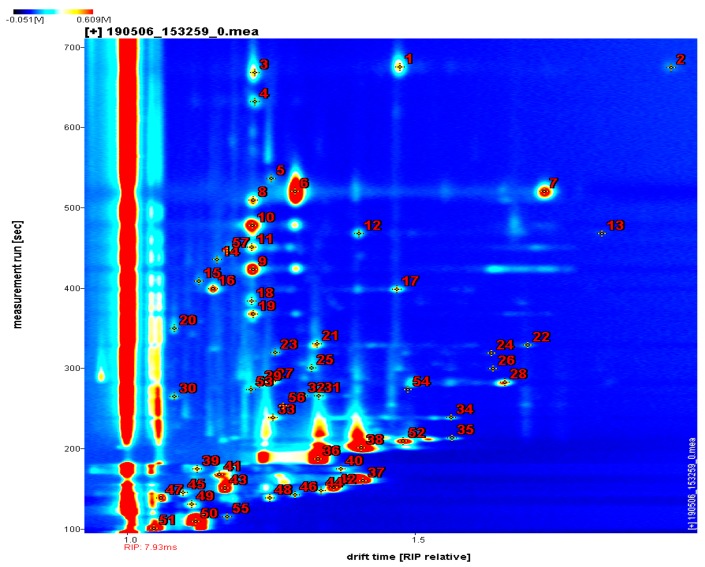
Ion migration spectra of volatile compounds represented by GC-IMS spectra in dry-cured beef. The numbers (1–57) are the identified volatile components.

The qualitative analysis of volatile components in the dry-cured beef is shown in Figure 4. The Y axis in the figure represents the retention time and the X axis represents the drift time. In the database, 57 peaks and 42 components were individually identified in this study, including 13 aldehydes, 11 alcohols, 6 ketones, 4 esters, 1 acid, and trimethyl pyrazine.

The compound name, CAS number, molecular formula, molecular mass, retention index, retention time, and drift time are listed in Table 2. As product ions pass through the drift region, adducts formed between the analyzed ions and neutral molecules (such as dimers and trimers), where the high mobility of a single compound resulted in multiple signals being observed [34]. Because of the presence of monomers and dimers, the compounds of nonanal, eucalyptol, octanal, benzaldehyde, heptanal, 2-heptanone, 1-hexanol, ethyl 3-methylbutanoate, furfurol, hexanal, 2-pentanone, isobutanol, ethyl acetate, 2-butanone, and 3-methyl butanoic acid appeared in two peaks.

**Figure 5:**
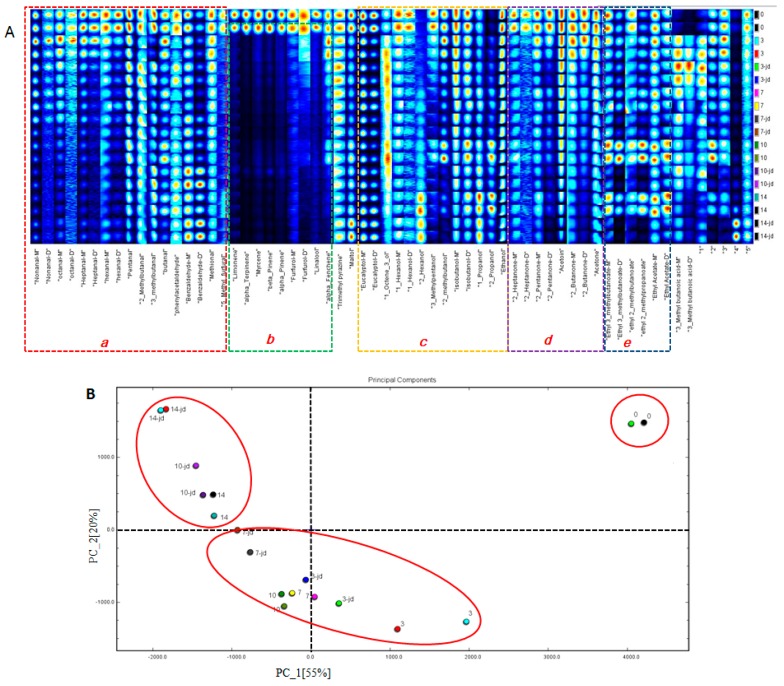
(**A**) The gallery plots of volatile compounds in dry-cured beef samples at 0, 3, 7, 10, and 14 days under different treatments. Each column represents the same substance in different samples, and each row represents a sample. a, red frame; b, green frame; c, yellow frame; d, purple frame; e, blue frame. (**B**) Principal component analysis (PCA) scatter diagram of volatile compounds of dry-cured beef samples.

Figure 5A shows a gallery plot of each sample and their differences in terms of color. The more pronounced color indicates higher VOCs. The numbers refer to an unidentified substance. The VOCs of the dry-cured beef constantly changed during storage. The materials in the red frame, such as nonanal, octana, heptanal, hexanal, pentanal, 2-methylbutanal, 3-methylbutanal, butanal, phenylacetaldehyde, benzaldehyde, methional, and 5-methyl-furfural, changed with the extension of the storage time. The main volatile compounds in meat were aldehydes [35,36]. As can be seen, at 10 and 14 days, the concentration of benzaldehyde in beef treated with HVEF was higher than that in the CON group. Benzaldehyde, known to contribute to a cherry-like odor [37], is a volatile compound potentially resulting from the Strecker degradation of tyrosine [38]. Substances in the green-framed areas, such as limonene, alpha-terpinene, myrcene, beta-pinene, alpha-pinene, furfurol linalool, and alpha-fenchene, were the most abundant on day 0. However, these substances appeared to decrease significantly over time. Ten alcohol-based compounds were identified in the yellow frame, including eucalyptol, 1-octene-3-ol, 1-hexanol, 2-hexanol, 3-methylpentanol, 2-methylbutanol, isobutanol, 1-propanol, 2-propano, and ethanol. Especially, 1-octen-3-ol was a secondary product from aldehydes and would produce mushroom odours [39]. The amount of 1-octen-3-ol in HVEF group on day 3 and in the CON group on day 10 showed a similar brightness. The content of acetoin (3-hydroxy-2-butanone) gradually creased with the extension of the storage time in the purple frame. Previous study demonstrated that acetoin was associated with consumer overall liking and flavor preference [40]. In this study, acetoin was only affected by the storage time. In the blue frame, the concentration of esters (ethyl 3-methylbutanoate, ethyl 2-methylbutanoate, ethyl 2-methylpropanoate, ethyl acetate) in control group was higher than that in HVEF group on days 10 and 14. The esters are formed by esterification reactions between ethanol and carboxylic acids and usually characterized by sweet and fruity odors [41,42]. One prior work has reported that esters production might be linked to the presence of certain microorganisms [43]. Some typical heterocyclic compounds in HVEF group significantly increased after 14 days, such as trimethyl pyrazine and maltol, which possessed low odor thresholds [37] and enhanced the characteristic flavor of the dry-cured beef.

To better assess the relationship between volatile compounds in HVEF treatment and time, principal component analysis (PCA) was applied to the volatile data (Figure 5B). The results indicated that the first two principal components explain 75% of the total variance (55% and 20%, respectively). The results clearly showed that storage time had a greater effect on the dry-cured beef than treatment, and that the distance between day 0 and the other samples was largely due to the composition of the volatile compounds. There was a higher similarity between the samples on day 10 in the HVEF group and the samples on day 14 in the CON group. The samples on day 3 of the HVEF group and the samples on day 7 and day 10 of the CON group were closer, demonstrating that the VOCs in these treatments were similar. This study also proved that HVEF had a positive effect on the flavor of dry-cured beef. 

### 3.5. Industrial Relevance

Under actual production conditions, HVEF technology was widely used to thaw meat in the food industry. This research demonstrated that HVEF had a positive effect on improving the color stability and enhancing flavor of dry-cured beef during storage. It therefore provided valuable information about the absolute limits of HVEF for the quality of dry-cured meat. However, there are some obvious limitations in practical applications. The output voltage and energy consumption of HVEF are very high, which is difficult to apply in large-scale meat processing for safety reasons.

## 4. Conclusions

In this research, the HVEF treatment delayed the decrease in pH and moisture content of dry-cured beef. HVEF treatment could keep the dry-cured beef with a higher color stability than the normal storage condition. In addition, the HVEF treatment could increase benzaldehyde concentrations, which might greatly influence the final dry-cured beef flavor. Further research should be assessed to reveal the mechanism of this process and to explore the impact of HVEF on the protein oxidation of dry-cured meat.

## Figures and Tables

**Table 1:** Influence of HVEF on color parameters of dry-cured beef during storage.

Item	Treatments (T)	Days (D)					SEM ^1^	Significance		
		0	3	7	10	14		T	D	T × D
*L* *	CON	33.15 ^a,b^	33.41 ^a^	33.47 ^a^	31.82 ^a,b^	31.41 ^b^	0.552	NS	**	NS
	HVEF	33.07 ^a,b^	33.03 ^a,b^	33.83 ^a^	31.94 ^b^	32.47 ^a,b^				
*a* *	CON	18.45 ^a^	15.89 ^b^	14.59 ^b,c^	13.84 ^c^	13.14 ^c^	0.402	NS	***	NS
	HVEF	17.17 ^a^	15.88 ^a,b^	15.26 ^b^	13.6 ^c^	12.81 ^c^				
*b* *	CON	9.28 ^a^	9.32 ^a^	8.53 ^a,b^	7.57 ^a,b^	7.31 ^b^	0.471	NS	***	NS
	HVEF	9.55 ^a^	9.81 ^a^	9.69 ^a^	7.95 ^b^	8.01 ^b^				
*C* *	CON	20.66 ^a^	18.44 ^b^	16.95 ^b,c^	15.81 ^c,d^	15.09 ^d^	0.511	NS	***	NS
	HVEF	19.66 ^a^	18.69 ^a^	18.10 ^a^	15.79 ^b^	15.15 ^b^				
∆*E*	CON	-	3.33 ^b^	4.97 ^a,A^	5.75 ^a^	6.47 ^a^	0.554	***	***	NS
	HVEF	-	2.11 ^c^	2.53 ^c,B^	4.23 ^a,b^	5.03 ^a^				

^a–c^ Averages with different letters in the same row are significantly different (*p* < 0.05). ^A,B^ Averages with different letters in the same column are significantly different (*p* < 0.05). NS, not significant; *, *p* < 0.05; **, *p* < 0.01; ***, *p* < 0.001. ^1^ SEM, standard error of the mean.

**Table 2:** The information on identified compounds of dry-cured beef during storage.

Number	Compound	CAS ^#^	Formula	Molecular Mass	Retention Index	Retention Time (s)	Drift Time (ms)
1	Nonanal (Monomer)	C124196	C9H18O	142.2	1104.8	674.937	1.4764
2	Nonanal (Dimer)	C124196	C9H18O	142.2	1104.4	674.07	1.9482
3	Maltol	C118718	C6H6O3	126.1	1101.6	668.002	1.2237
4	Linalool	C78706	C10H18O	154.3	1085.3	631.593	1.2237
5	Phenylacetaldehyde	C122781	C8H8O	120.2	1041	536.148	1.2517
6	Eucalyptol (Monomer)	C470826	C10H18O	154.3	1033.1	520.193	1.293
7	Eucalyptol (Dimer)	C470826	C10H18O	154.3	1033.1	520.193	1.7271
8	Limonene	C138863	C10H16	136.2	1027.5	508.891	1.2207
9	Beta-Pinene	C127913	C10H16	136.2	978.4	423.131	1.2207
10	Alpha-Terpinene	C99865	C10H16	136.2	1011	477.646	1.2192
11	Myrcene	C123353	C10H16	136.2	995.5	450.388	1.2178
12	Octanal (Monomer)	C124130	C8H16O	128.2	1005.5	467.673	1.4038
13	Octanal (Dimer)	C124130	C8H16O	128.2	1005.5	467.673	1.8275
14	1-Octene-3-ol	C3391864	C8H16O	128.2	986.2	435.098	1.1572
15	5-Methyl-furfural	C620020	C6H6O2	110.1	968	407.841	1.1262
16	Benzaldehyde (Monomer)	C100527	C7H6O	106.1	961.7	399.198	1.1513
17	Benzaldehyde (Dimer)	C100527	C7H6O	106.1	961.3	398.534	1.4702
18	Alpha-Fenchene	C471841	C10H16	136.2	949.7	383.243	1.2178
19	Alpha-Pinene	C80568	C10H16	136.2	936.8	367.288	1.2207
20	Methional	C3268493	C4H8OS	104.2	921	349.338	1.0834
21	Heptanal (Monomer)	C111717	C7H14O	114.2	902.6	330.059	1.3314
22	Heptanal (Dimer)	C111717	C7H14O	114.2	901.2	328.729	1.6976
23	2-Heptanone (Monomer)	C110430	C7H14O	114.2	892	319.736	1.2594
24	2-Heptanone (Dimer)	C110430	C7H14O	114.2	890.9	318.733	1.6345
25	1-Hexanol (Monomer)	C111273	C6H14O	102.2	870.4	300.009	1.3219
26	1-Hexanol (Dimer)	C111273	C6H14O	102.2	870	299.675	1.6382
27	Ethyl 3-methylbutanoate (Monomer)	C108645	C7H14O2	130.2	850.7	283.291	1.2519
28	Ethyl 3-methylbutanoate (Dimer)	C108645	C7H14O2	130.2	849.9	282.623	1.6582
29	Ethyl 2-methylbutanoate	C7452791	C7H14O2	130.2	846.6	279.948	1.2319
30	Furfurol (Monomer)	C98011	C5H4O2	96.1	827.6	264.902	1.0831
31	Furfurol	C98011	C5H4O2	96.1	828.1	265.236	1.3344
32	3-Methylpentanol	C589355	C6H14O	102.2	828.9	265.905	1.3069
33	Hexanal (Monomer)	C66251	C6H12O	100.2	792.5	238.822	1.2544
34	Hexanal (Dimer)	C66251	C6H12O	100.2	792.1	238.488	1.5644
35	Ethyl 2-methylpropanoate	C97621	C6H12O2	116.2	755.6	213	1.5669
36	Acetoin	C513860	C4H8O2	88.1	713.8	187.134	1.3336
37	3-Methylbutanal	C590863	C5H10O	86.1	653.9	160.055	1.4114
38	Pentanal	C110623	C5H10O	86.1	736.6	200.673	1.4084
39	2-Pentanone (Monomer)	C107879	C5H10O	86.1	689.1	174.403	1.1237
40	2-Pentanone (Dimer)	C107879	C5H10O	86.1	689.1	174.403	1.3735
41	2-Methylbutanal	C96173	C5H10O	86.1	672.9	167.33	1.1616
42	Isobutanol (Dimer)	C78831	C4H10O	74.1	629.2	151.77	1.3608
43	Isobutanol (Monomer)	C78831	C4H10O	74.1	626.6	150.962	1.1713
44	Ethyl Acetate (Dimer)	C141786	C4H8O2	88.1	614.7	147.324	1.3385
45	Ethyl Acetate (Monomer)	C141786	C4H8O2	88.1	607.2	145.101	1.0984
46	Butanal	C123728	C4H8O	72.1	596.9	142.07	1.2928
47	2-Butanone (Monomer)	C78933	C4H8O	72.1	584.3	138.433	1.0605
48	2-Butanone (Dimer)	C78933	C4H8O	72.1	584.3	138.433	1.2491
49	1-Propanol	C71238	C3H8O	60.1	557.7	130.754	1.114
50	Acetone	C67641	C3H6O	58.1	482.3	108.93	1.1208
51	Ethanol	C64175	C2H6O	46.1	453.7	100.644	1.0479
52	2-Methylbutanol	C137326	C5H12O	88.1	749.5	208.939	1.4811
53	3-Methyl butanoic acid (Monomer)	C503742	C5H10O2	102.1	838.7	273.567	1.2168
54	3-Methyl butanoic acid (Dimer)	C503742	C5H10O2	102.1	838.2	273.158	1.4899
55	2-Propanol	C67630	C3H8O	60.1	503	114.909	1.1753
56	2-Hexanol	C626937	C6H14O	102.2	812	253.093	1.2727
57	Trimethyl pyrazine	C14667551	C7H10N2	122.2	993	446.225	1.1757

^#^ CAS, the registration number of chemical substances.
